# *fac*-Tris(4-amino­benzohydroxamato)iron(III) ethanol solvate

**DOI:** 10.1107/S160053681002413X

**Published:** 2010-06-26

**Authors:** Ahmed B. M. Alagha, Declan Gaynor, Helge Müller-Bunz, Kevin B. Nolan, Laavanya Parthasarathi

**Affiliations:** a Centre for Synthesis and Chemical Biology, Department of Pharmaceutical and Medicinal Chemistry, Royal College of Surgeons in Ireland, Dublin 2, Ireland; b Centre for Synthesis and Chemical Biology, School of Chemistry and Chemical Biology, University College Dublin, Dublin 4, Ireland; c Smurfit Institute of Genetics, Trinity College Dublin, Dublin 2, Ireland

## Abstract

In the structure of the title compound, [Fe(C_7_H_7_N_2_O_2_)_3_]·CH_3_CH_2_OH, the Fe^III^ atom is in a distorted octa­hedral O_6_ environment with the three hydroxamate O atoms (and the three carbonyl O atoms) arranged in a *fac* configuration and one of the hydroxamate ligands being puckered. The methyl C atom of the ethanol solvent mol­ecule is disordered over two positions with occupancies of 0.626 (13) and 0.374 (13), respectively. The cocrystallized ethanol mol­ecule is hydrogen bonded to one of the hydroxamate O atoms. O—H⋯O and N—H⋯O inter­actions generate infinite three-dimensional networks along [100], [010] and [001].

## Related literature

For a detailed account of the mol­ecular and crystal structures of related tris­(hydroxamato)Fe^III^ complexes, see: Rio-Echevarria *et al.* (2008 ▶); Mulcahy *et al.* (2007 ▶); Marmion *et al.* (2000 ▶). For ring puckering parameters, see: Cremer & Pople (1975 ▶); for pseudorotation parameters, see: Rao *et al.* (1981 ▶).
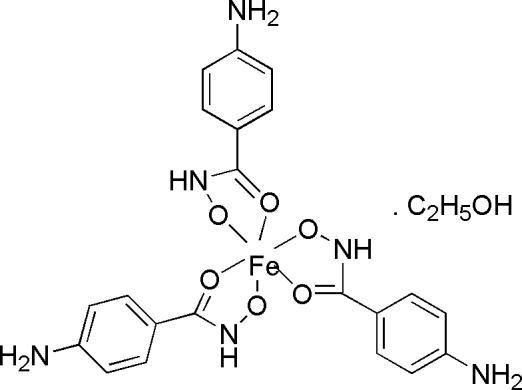


## Experimental

### 

#### Crystal data

[Fe(C_7_H_7_N_2_O_2_)_3_]·C_2_H_6_O
*M* *_r_* = 555.36
Triclinic, 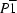

*a* = 10.6232 (18) Å
*b* = 10.6455 (18) Å
*c* = 13.225 (2) Å
α = 107.550 (3)°
β = 91.085 (4)°
γ = 112.217 (3)°
*V* = 1305.1 (4) Å^3^
*Z* = 2
Mo *K*α radiation
μ = 0.63 mm^−1^
*T* = 100 K
0.30 × 0.20 × 0.03 mm

#### Data collection

Bruker SMART CCD area detector diffractometer
Absorption correction: multi-scan (*SADABS*; Sheldrick, 2003 ▶) *T*_min_ = 0.883, *T*_max_ = 0.981
24614 measured reflections
5739 independent reflections
5065 reflections with *I* > 2σ(*I*)
*R*_int_ = 0.044

#### Refinement

*R*[*F* ^2^ > 2σ(*F* ^2^)] = 0.040
*wR*(*F* ^2^) = 0.094
*S* = 1.06
5739 reflections
433 parameters
H atoms treated by a mixture of independent and constrained refinement
Δρ_max_ = 0.43 e Å^−3^
Δρ_min_ = −0.31 e Å^−3^

### 

Data collection: *SMART* (Bruker, 2003 ▶); cell refinement: *SAINT* (Bruker, 2003 ▶); data reduction: *SAINT*; program(s) used to solve structure: *SHELXS97* (Sheldrick, 2008 ▶); program(s) used to refine structure: *SHELXL97* (Sheldrick, 2008 ▶); molecular graphics: *PLATON* (Spek, 2009 ▶); software used to prepare material for publication: *SHELXL97*.

## Supplementary Material

Crystal structure: contains datablocks I, global. DOI: 10.1107/S160053681002413X/sj5023sup1.cif

Structure factors: contains datablocks I. DOI: 10.1107/S160053681002413X/sj5023Isup2.hkl

Additional supplementary materials:  crystallographic information; 3D view; checkCIF report

## Figures and Tables

**Table 1:** Selected bond lengths (Å)

Fe—O2	1.9615 (14)
Fe—O6	1.9769 (14)
Fe—O4	1.9822 (14)
Fe—O5	2.0444 (14)
Fe—O1	2.0470 (14)
Fe—O3	2.0654 (13)

**Table 2:** Hydrogen-bond geometry (Å, °)

*D*—H⋯*A*	*D*—H	H⋯*A*	*D*⋯*A*	*D*—H⋯*A*
O7—H1*O*7⋯O6^i^	0.78 (3)	1.91 (3)	2.694 (2)	175 (3)
N6—H1*N*6⋯O3^ii^	0.84 (2)	2.15 (2)	2.968 (2)	163.9 (19)
N5—H1*N*5⋯O7	0.84 (2)	1.91 (2)	2.740 (2)	176 (2)
N3—H1*N*3⋯O4^iii^	0.86 (3)	1.98 (3)	2.755 (2)	150 (2)
N1—H1*N*1⋯O2^iv^	0.84 (3)	1.99 (3)	2.737 (2)	149 (2)

Symmetry codes: (i) 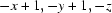; (ii) 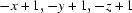; (iii) 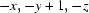; (iv) 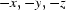.

**Fig. 1.**
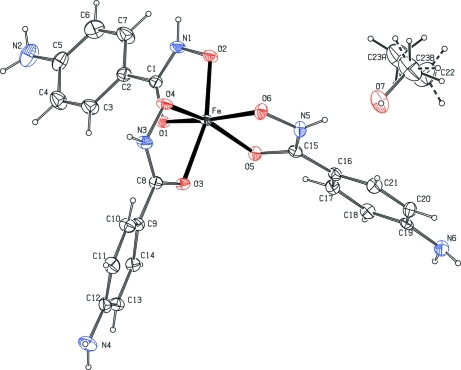
The asymmetric unit of (I) with the atoms labelled and displacement ellipsoids depicted at the 50% probability level for all non-H atoms. H-atoms are drawn as spheres of arbitrary radius

**Fig. 2.**
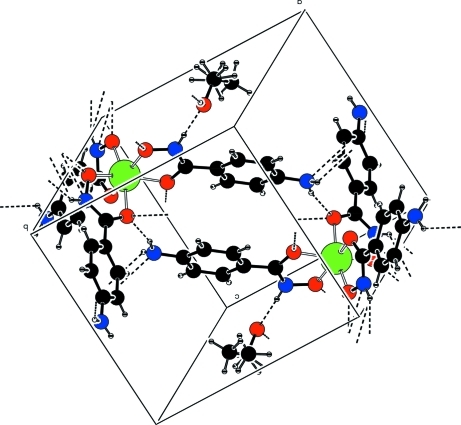
The molecular packing viewed down the b-axis. Dashed lines represent the O—H···O and N—H···O interactions within the lattice.

## supplementary crystallographic information

### Comment

The Fe^III^ core is in a distorted octahedral O6 environment with the three 4-aminophenylhydroxamato ligands coordinated through both O-atoms to form stable five-membered chelates (Fig. 1). The complex has a *fac* geometric configuration defined by the positioning of the three hydroxamate or the three carbonyl O atoms. The Fe—O3—C8—N3—O4 and Fe—O5—C15—N5—O6 five membered rings are not puckered whereas the Fe—O1—C1—N1—O2 ring exhibits a puckered configuration with the closest pucker descriptor being twisted on O2—Fe. The Cremer and Pople puckering paramaters (Cremer & Pople, 1975) are Q2 = 0.120 (2) Å & φ2 = 330.0 (9)° and the pseudorotation parameters (Rao *et al.*,  1981) are P = 130.8 (6)° and τ(*M*) = 11.2 (1)° for reference bond Fe—O1. The co-crystallized ethanol molecule is hydrogen bonded to one of the hydroxamate oxygen atoms through strong O–H···O bond (O7 H1O7 O6 interaction in Table 2). The O–H···O and N–H···O interactions (Table 2) form a set of clusters generating infinite two dimensional networks along the base vectors [0 1 0] and [1 0 0] and along the plane (0 0 1). These clusters then assemble to form infinite three dimensional networks (Fig. 2) along the base vectors [1 0 0], [0 1 0] and [0 0 1].

### Experimental

FeCl_3_.6H_2_O (59.3 mg, 0.22 mmol) in water (5 ml), was added to a solution of 4-aminophenylhydroxamic acid, (100 mg, 0.66 mmol) in ethanol (15 ml). The pH of the resulting solution was then raised to 5.7 using 0.1 *M* NaOH solution whereupon a dark red precipitate was obtained. This was removed by filtration and the filtrate was left to stand at room temperature for two months whereupon very dark red crystals were obtained. These were collected by filtration and dried. Yield (100 mg, 0.18 mmol, 82%). Elemental analysis (%), calcd C_23_H_27_N_6_O_7_Fe: C, 49.56; H, 5.26; N, 15.08; Fe, 10.02; found C 49.01, H 5.02, N 15.03, Fe 9.77. IR (KBr Disc); ν_max_ 3345br, 3211br, 1604 s, 1525 s, 1484 s cm^-1^.

### Refinement

All H-atoms (except those attached to the methylene and the methyl carbon atoms of the ethanol solvate) were located in difference maps and their positions and isotropic displacement parameters were freely refined. The CH_2_ and CH_3_ H atoms of the solvate were constrained to ride on their parent C atoms.

### Crystal data

**Table:**

[Fe(C_7_H_7_N_2_O_2_)_3_]·C_2_H_6_O	*Z* = 2
*M**_r_* = 555.36	*F*(000) = 578
Triclinic, *P*1	*D*_x_ = 1.413 Mg m^−^^3^
Hall symbol: -P 1	Mo *K*α radiation, λ = 0.71073 Å
*a* = 10.6232 (18) Å	Cell parameters from 4743 reflections
*b* = 10.6455 (18) Å	θ = 2.2–24.9°
*c* = 13.225 (2) Å	µ = 0.63 mm^−^^1^
α = 107.550 (3)°	*T* = 100 K
β = 91.085 (4)°	Plate, red
γ = 112.217 (3)°	0.30 × 0.20 × 0.03 mm
*V* = 1305.1 (4) Å^3^	

### Data collection

**Table:**

Bruker SMART CCD area detector diffractometer	5739 independent reflections
Radiation source: fine-focus sealed tube	5065 reflections with *I* > 2σ(*I*)
graphite	*R*_int_ = 0.044
Detector resolution: 8.366 pixels mm^-1^	θ_max_ = 27.1°, θ_min_ = 1.6°
φ and ω scans	*h* = −13→13
Absorption correction: multi-scan (*SADABS*; Sheldrick, 2003)	*k* = −13→13
*T*_min_ = 0.883, *T*_max_ = 0.981	*l* = −16→16
24614 measured reflections	

### Refinement

**Table:**

Refinement on *F*^2^	Primary atom site location: structure-invariant direct methods
Least-squares matrix: full	Secondary atom site location: difference Fourier map
*R*[*F*^2^ > 2σ(*F*^2^)] = 0.040	Hydrogen site location: difference Fourier map
*wR*(*F*^2^) = 0.094	H atoms treated by a mixture of independent and constrained refinement
*S* = 1.06	*w* = 1/[σ^2^(*F*_o_^2^) + (0.0438*P*)^2^ + 0.6552*P*] where *P* = (*F*_o_^2^ + 2*F*_c_^2^)/3
5739 reflections	(Δ/σ)_max_ = 0.004
433 parameters	Δρ_max_ = 0.43 e Å^−^^3^
0 restraints	Δρ_min_ = −0.31 e Å^−^^3^

### Special details

**Table:**

Geometry. All e.s.d.'s (except the e.s.d. in the dihedral angle between two l.s. planes) are estimated using the full covariance matrix. The cell e.s.d.'s are taken into account individually in the estimation of e.s.d.'s in distances, angles and torsion angles; correlations between e.s.d.'s in cell parameters are only used when they are defined by crystal symmetry. An approximate (isotropic) treatment of cell e.s.d.'s is used for estimating e.s.d.'s involving l.s. planes.
Refinement. Refinement of *F*^2^ against ALL reflections. The weighted *R*-factor *wR* and goodness of fit *S* are based on *F*^2^, conventional *R*-factors *R* are based on *F*, with *F* set to zero for negative *F*^2^. The threshold expression of *F*^2^ > σ(*F*^2^) is used only for calculating *R*-factors(gt) *etc*. and is not relevant to the choice of reflections for refinement. *R*-factors based on *F*^2^ are statistically about twice as large as those based on *F*, and *R*- factors based on ALL data will be even larger.

### Fractional atomic coordinates and isotropic or equivalent isotropic displacement parameters (Å^2^)

**Table:**

	*x*	*y*	*z*	*U*_iso_*/*U*_eq_	Occ. (<1)
Fe	0.13879 (3)	0.36163 (3)	0.16284 (2)	0.01220 (9)	
O2	0.06647 (14)	0.16143 (14)	0.06621 (11)	0.0180 (3)	
N1	−0.05305 (18)	0.07800 (18)	0.09477 (14)	0.0187 (4)	
H1N1	−0.087 (3)	−0.007 (3)	0.052 (2)	0.035 (7)*	
C1	−0.0862 (2)	0.1334 (2)	0.18804 (16)	0.0161 (4)	
O1	−0.01237 (14)	0.26557 (14)	0.24206 (11)	0.0162 (3)	
C2	−0.2049 (2)	0.0445 (2)	0.22618 (16)	0.0179 (4)	
C3	−0.2669 (2)	0.1117 (2)	0.30398 (18)	0.0239 (5)	
H3	−0.231 (3)	0.211 (3)	0.331 (2)	0.039 (7)*	
C4	−0.3816 (2)	0.0325 (3)	0.3394 (2)	0.0279 (5)	
H4	−0.425 (3)	0.078 (3)	0.391 (2)	0.031 (7)*	
C5	−0.4376 (2)	−0.1172 (2)	0.29942 (18)	0.0243 (5)	
N2	−0.5509 (2)	−0.1970 (3)	0.3375 (2)	0.0340 (5)	
H1N2	−0.595 (3)	−0.277 (3)	0.296 (2)	0.040 (9)*	
H2N2	−0.602 (3)	−0.147 (3)	0.364 (2)	0.048 (9)*	
C6	−0.3739 (3)	−0.1848 (2)	0.2234 (2)	0.0317 (5)	
H6	−0.410 (3)	−0.288 (3)	0.198 (2)	0.043 (8)*	
C7	−0.2604 (2)	−0.1056 (2)	0.1865 (2)	0.0286 (5)	
H7	−0.219 (3)	−0.152 (3)	0.136 (2)	0.037 (7)*	
O4	0.04007 (15)	0.41933 (14)	0.06861 (11)	0.0175 (3)	
N3	0.03983 (18)	0.55267 (18)	0.11997 (14)	0.0188 (4)	
H1N3	0.008 (3)	0.585 (3)	0.078 (2)	0.029 (7)*	
C8	0.1040 (2)	0.6232 (2)	0.21800 (15)	0.0146 (4)	
O3	0.15716 (14)	0.56033 (14)	0.26357 (10)	0.0149 (3)	
C9	0.1150 (2)	0.7704 (2)	0.27167 (16)	0.0154 (4)	
C10	0.1173 (2)	0.8613 (2)	0.21373 (17)	0.0196 (4)	
H10	0.116 (2)	0.832 (2)	0.1403 (18)	0.014 (5)*	
C11	0.1315 (2)	1.0008 (2)	0.26579 (17)	0.0205 (4)	
H11	0.135 (2)	1.060 (2)	0.2244 (17)	0.015 (5)*	
C12	0.1403 (2)	1.0527 (2)	0.37769 (17)	0.0184 (4)	
N4	0.1479 (2)	1.1887 (2)	0.42768 (17)	0.0238 (4)	
H1N4	0.165 (3)	1.217 (3)	0.494 (2)	0.027 (7)*	
H2N4	0.161 (3)	1.247 (3)	0.388 (2)	0.042 (8)*	
C13	0.1409 (2)	0.9625 (2)	0.43591 (17)	0.0182 (4)	
H13	0.148 (2)	0.995 (3)	0.508 (2)	0.024 (6)*	
C14	0.1295 (2)	0.8241 (2)	0.38380 (16)	0.0168 (4)	
H14	0.129 (2)	0.765 (2)	0.4247 (17)	0.010 (5)*	
O6	0.30277 (14)	0.41024 (15)	0.09015 (11)	0.0178 (3)	
N5	0.41597 (18)	0.41538 (19)	0.14684 (14)	0.0173 (4)	
H1N5	0.480 (2)	0.412 (2)	0.1112 (18)	0.016 (6)*	
C15	0.4028 (2)	0.3985 (2)	0.24097 (15)	0.0145 (4)	
O5	0.28464 (14)	0.37299 (14)	0.27232 (11)	0.0157 (3)	
C16	0.5226 (2)	0.4146 (2)	0.30955 (15)	0.0154 (4)	
C17	0.5013 (2)	0.3612 (2)	0.39495 (16)	0.0178 (4)	
H17	0.408 (2)	0.311 (2)	0.4045 (18)	0.021 (6)*	
C18	0.6104 (2)	0.3822 (2)	0.46526 (16)	0.0175 (4)	
H18	0.592 (2)	0.346 (3)	0.5211 (19)	0.024 (6)*	
C19	0.7457 (2)	0.4587 (2)	0.45299 (15)	0.0158 (4)	
N6	0.8551 (2)	0.4808 (2)	0.52404 (15)	0.0198 (4)	
H1N6	0.835 (2)	0.467 (2)	0.5823 (18)	0.008 (5)*	
H2N6	0.927 (3)	0.549 (3)	0.529 (2)	0.037 (8)*	
C20	0.7675 (2)	0.5081 (2)	0.36565 (17)	0.0186 (4)	
H20	0.854 (2)	0.559 (2)	0.3568 (17)	0.015 (5)*	
C21	0.6581 (2)	0.4880 (2)	0.29572 (17)	0.0187 (4)	
H21	0.677 (2)	0.527 (2)	0.2400 (17)	0.012 (5)*	
O7	0.62865 (18)	0.41913 (19)	0.03203 (14)	0.0300 (4)	
H1O7	0.651 (3)	0.473 (3)	0.000 (2)	0.030 (7)*	
C22	0.6632 (3)	0.2992 (3)	−0.0101 (2)	0.0450 (7)	
H22A	0.7633	0.3304	0.0097	0.054*	0.626 (13)
H22B	0.6425	0.2649	−0.0893	0.054*	0.626 (13)
H22C	0.5911	0.2283	−0.0711	0.054*	0.374 (13)
H22D	0.7502	0.3321	−0.0395	0.054*	0.374 (13)
C23A	0.5904 (9)	0.1809 (6)	0.0278 (5)	0.048 (2)	0.626 (13)
H23A	0.5992	0.2168	0.1062	0.073*	0.626 (13)
H23B	0.6297	0.1088	0.0061	0.073*	0.626 (13)
H23C	0.4929	0.1371	−0.0032	0.073*	0.626 (13)
C23B	0.6799 (14)	0.2255 (11)	0.0608 (8)	0.052 (3)	0.374 (13)
H23D	0.7001	0.1432	0.0207	0.077*	0.374 (13)
H23E	0.5950	0.1914	0.0912	0.077*	0.374 (13)
H23F	0.7561	0.2917	0.1189	0.077*	0.374 (13)

### Atomic displacement parameters (Å^2^)

**Table:**

	*U*^11^	*U*^22^	*U*^33^	*U*^12^	*U*^13^	*U*^23^
Fe	0.01437 (15)	0.00964 (14)	0.01186 (14)	0.00477 (11)	0.00097 (10)	0.00273 (10)
O2	0.0186 (7)	0.0124 (7)	0.0189 (7)	0.0040 (6)	0.0068 (6)	0.0023 (6)
N1	0.0189 (9)	0.0108 (8)	0.0199 (9)	0.0027 (7)	0.0037 (7)	0.0002 (7)
C1	0.0155 (9)	0.0125 (9)	0.0187 (10)	0.0061 (8)	0.0006 (8)	0.0028 (8)
O1	0.0176 (7)	0.0102 (6)	0.0168 (7)	0.0036 (5)	0.0032 (5)	0.0015 (5)
C2	0.0178 (10)	0.0148 (10)	0.0201 (10)	0.0057 (8)	0.0022 (8)	0.0058 (8)
C3	0.0252 (11)	0.0143 (10)	0.0286 (12)	0.0054 (9)	0.0085 (9)	0.0052 (9)
C4	0.0281 (12)	0.0259 (12)	0.0317 (13)	0.0118 (10)	0.0146 (10)	0.0106 (10)
C5	0.0181 (10)	0.0253 (11)	0.0301 (12)	0.0043 (9)	0.0001 (9)	0.0161 (10)
N2	0.0229 (11)	0.0333 (13)	0.0448 (14)	0.0024 (10)	0.0056 (10)	0.0229 (11)
C6	0.0296 (13)	0.0151 (11)	0.0439 (15)	0.0021 (10)	0.0070 (11)	0.0099 (10)
C7	0.0299 (12)	0.0150 (10)	0.0382 (14)	0.0087 (9)	0.0126 (11)	0.0051 (10)
O4	0.0266 (8)	0.0127 (7)	0.0132 (7)	0.0111 (6)	−0.0023 (6)	0.0004 (5)
N3	0.0263 (9)	0.0166 (9)	0.0158 (9)	0.0130 (7)	−0.0014 (7)	0.0033 (7)
C8	0.0161 (9)	0.0137 (9)	0.0142 (9)	0.0061 (8)	0.0024 (7)	0.0050 (7)
O3	0.0191 (7)	0.0115 (6)	0.0140 (7)	0.0063 (5)	−0.0011 (5)	0.0038 (5)
C9	0.0161 (9)	0.0143 (9)	0.0161 (9)	0.0073 (8)	0.0018 (7)	0.0041 (8)
C10	0.0257 (11)	0.0190 (10)	0.0162 (10)	0.0112 (9)	0.0029 (8)	0.0060 (8)
C11	0.0253 (11)	0.0186 (10)	0.0231 (11)	0.0117 (9)	0.0041 (9)	0.0108 (9)
C12	0.0146 (9)	0.0151 (10)	0.0256 (11)	0.0063 (8)	0.0057 (8)	0.0065 (8)
N4	0.0326 (11)	0.0153 (9)	0.0239 (11)	0.0109 (8)	0.0077 (8)	0.0050 (8)
C13	0.0202 (10)	0.0168 (10)	0.0151 (10)	0.0065 (8)	0.0036 (8)	0.0032 (8)
C14	0.0185 (10)	0.0144 (9)	0.0181 (10)	0.0062 (8)	0.0028 (8)	0.0069 (8)
O6	0.0160 (7)	0.0245 (8)	0.0167 (7)	0.0092 (6)	0.0019 (6)	0.0105 (6)
N5	0.0152 (8)	0.0225 (9)	0.0181 (9)	0.0091 (7)	0.0045 (7)	0.0102 (7)
C15	0.0175 (10)	0.0103 (9)	0.0162 (9)	0.0063 (7)	0.0027 (8)	0.0042 (7)
O5	0.0158 (7)	0.0171 (7)	0.0160 (7)	0.0071 (6)	0.0025 (5)	0.0073 (6)
C16	0.0180 (10)	0.0141 (9)	0.0159 (9)	0.0086 (8)	0.0028 (8)	0.0046 (8)
C17	0.0181 (10)	0.0159 (10)	0.0202 (10)	0.0070 (8)	0.0052 (8)	0.0068 (8)
C18	0.0227 (10)	0.0196 (10)	0.0151 (10)	0.0109 (8)	0.0047 (8)	0.0094 (8)
C19	0.0195 (10)	0.0141 (9)	0.0141 (9)	0.0102 (8)	0.0022 (8)	0.0007 (7)
N6	0.0197 (10)	0.0247 (10)	0.0168 (9)	0.0100 (8)	0.0037 (7)	0.0083 (8)
C20	0.0142 (10)	0.0190 (10)	0.0209 (10)	0.0046 (8)	0.0045 (8)	0.0069 (8)
C21	0.0198 (10)	0.0203 (10)	0.0171 (10)	0.0069 (8)	0.0043 (8)	0.0092 (8)
O7	0.0367 (10)	0.0372 (10)	0.0326 (9)	0.0221 (8)	0.0197 (8)	0.0238 (8)
C22	0.0638 (19)	0.0503 (17)	0.0404 (16)	0.0374 (16)	0.0226 (14)	0.0222 (14)
C23A	0.063 (5)	0.036 (3)	0.058 (4)	0.026 (3)	0.021 (3)	0.023 (3)
C23B	0.048 (7)	0.039 (5)	0.069 (6)	0.022 (5)	−0.016 (5)	0.015 (4)

### Geometric parameters (Å, °)

**Table:**

Fe—O2	1.9615 (14)	N4—H1N4	0.83 (3)
Fe—O6	1.9769 (14)	N4—H2N4	0.90 (3)
Fe—O4	1.9822 (14)	C13—C14	1.382 (3)
Fe—O5	2.0444 (14)	C13—H13	0.90 (2)
Fe—O1	2.0470 (14)	C14—H14	0.94 (2)
Fe—O3	2.0654 (13)	O6—N5	1.379 (2)
O2—N1	1.383 (2)	N5—C15	1.313 (3)
N1—C1	1.315 (3)	N5—H1N5	0.84 (2)
N1—H1N1	0.84 (3)	C15—O5	1.285 (2)
C1—O1	1.282 (2)	C15—C16	1.474 (3)
C1—C2	1.466 (3)	C16—C17	1.396 (3)
C2—C3	1.394 (3)	C16—C21	1.400 (3)
C2—C7	1.396 (3)	C17—C18	1.377 (3)
C3—C4	1.378 (3)	C17—H17	0.96 (2)
C3—H3	0.93 (3)	C18—C19	1.400 (3)
C4—C5	1.391 (3)	C18—H18	0.92 (2)
C4—H4	0.94 (3)	C19—N6	1.381 (3)
C5—N2	1.390 (3)	C19—C20	1.396 (3)
C5—C6	1.395 (3)	N6—H1N6	0.84 (2)
N2—H1N2	0.80 (3)	N6—H2N6	0.82 (3)
N2—H2N2	0.90 (3)	C20—C21	1.380 (3)
C6—C7	1.380 (3)	C20—H20	0.90 (2)
C6—H6	0.96 (3)	C21—H21	0.94 (2)
C7—H7	0.92 (3)	O7—C22	1.417 (3)
O4—N3	1.378 (2)	O7—H1O7	0.78 (3)
N3—C8	1.304 (3)	C22—C23B	1.438 (8)
N3—H1N3	0.86 (3)	C22—C23A	1.446 (5)
C8—O3	1.283 (2)	C22—H22A	0.9900
C8—C9	1.470 (3)	C22—H22B	0.9900
C9—C10	1.398 (3)	C22—H22C	0.9900
C9—C14	1.401 (3)	C22—H22D	0.9900
C10—C11	1.382 (3)	C23A—H23A	0.9800
C10—H10	0.92 (2)	C23A—H23B	0.9800
C11—C12	1.402 (3)	C23A—H23C	0.9800
C11—H11	0.95 (2)	C23B—H23D	0.9800
C12—N4	1.368 (3)	C23B—H23E	0.9800
C12—C13	1.402 (3)	C23B—H23F	0.9800
			
O2—Fe—O6	90.72 (6)	C12—C13—H13	119.8 (15)
O2—Fe—O4	92.73 (6)	C13—C14—C9	120.68 (18)
O6—Fe—O4	91.70 (6)	C13—C14—H14	119.0 (13)
O2—Fe—O5	104.13 (6)	C9—C14—H14	120.3 (13)
O6—Fe—O5	78.92 (5)	N5—O6—Fe	112.13 (11)
O4—Fe—O5	160.68 (6)	C15—N5—O6	117.38 (16)
O2—Fe—O1	79.40 (5)	C15—N5—H1N5	127.0 (15)
O6—Fe—O1	162.99 (6)	O6—N5—H1N5	114.4 (15)
O4—Fe—O1	102.49 (6)	O5—C15—N5	118.19 (17)
O5—Fe—O1	90.01 (6)	O5—C15—C16	121.59 (17)
O2—Fe—O3	163.96 (6)	N5—C15—C16	120.18 (17)
O6—Fe—O3	102.91 (6)	C15—O5—Fe	113.19 (12)
O4—Fe—O3	78.63 (5)	C17—C16—C21	118.42 (18)
O5—Fe—O3	86.98 (5)	C17—C16—C15	119.09 (18)
O1—Fe—O3	89.21 (5)	C21—C16—C15	122.41 (18)
N1—O2—Fe	111.18 (11)	C18—C17—C16	121.01 (19)
C1—N1—O2	117.85 (16)	C18—C17—H17	120.6 (14)
C1—N1—H1N1	129.3 (18)	C16—C17—H17	118.4 (14)
O2—N1—H1N1	112.1 (18)	C17—C18—C19	120.58 (19)
O1—C1—N1	117.72 (18)	C17—C18—H18	118.3 (15)
O1—C1—C2	122.48 (17)	C19—C18—H18	121.1 (15)
N1—C1—C2	119.79 (17)	N6—C19—C20	120.91 (19)
C1—O1—Fe	112.64 (12)	N6—C19—C18	120.57 (19)
C3—C2—C7	118.2 (2)	C20—C19—C18	118.49 (18)
C3—C2—C1	119.10 (18)	C19—N6—H1N6	115.4 (14)
C7—C2—C1	122.69 (19)	C19—N6—H2N6	117 (2)
C4—C3—C2	121.1 (2)	H1N6—N6—H2N6	115 (2)
C4—C3—H3	119.6 (17)	C21—C20—C19	120.82 (19)
C2—C3—H3	119.3 (17)	C21—C20—H20	119.1 (14)
C3—C4—C5	120.8 (2)	C19—C20—H20	119.9 (14)
C3—C4—H4	121.3 (16)	C20—C21—C16	120.62 (19)
C5—C4—H4	117.9 (16)	C20—C21—H21	118.1 (13)
N2—C5—C4	120.7 (2)	C16—C21—H21	121.3 (13)
N2—C5—C6	121.0 (2)	C22—O7—H1O7	113 (2)
C4—C5—C6	118.3 (2)	O7—C22—C23B	118.2 (4)
C5—N2—H1N2	114 (2)	O7—C22—C23A	113.5 (3)
C5—N2—H2N2	112.1 (19)	C23B—C22—C23A	37.0 (4)
H1N2—N2—H2N2	114 (3)	O7—C22—H22A	108.9
C7—C6—C5	121.0 (2)	C23B—C22—H22A	73.0
C7—C6—H6	120.4 (17)	C23A—C22—H22A	108.9
C5—C6—H6	118.6 (17)	O7—C22—H22B	108.9
C6—C7—C2	120.6 (2)	C23B—C22—H22B	129.9
C6—C7—H7	120.1 (17)	C23A—C22—H22B	108.9
C2—C7—H7	119.2 (17)	H22A—C22—H22B	107.7
N3—O4—Fe	111.77 (11)	O7—C22—H22C	107.7
C8—N3—O4	118.68 (16)	C23B—C22—H22C	107.9
C8—N3—H1N3	127.3 (17)	C23A—C22—H22C	75.6
O4—N3—H1N3	113.2 (17)	H22A—C22—H22C	136.9
O3—C8—N3	117.74 (17)	H22B—C22—H22C	37.7
O3—C8—C9	122.39 (17)	O7—C22—H22D	107.7
N3—C8—C9	119.87 (17)	C23B—C22—H22D	107.6
C8—O3—Fe	113.04 (12)	C23A—C22—H22D	135.6
C10—C9—C14	118.69 (18)	H22A—C22—H22D	39.5
C10—C9—C8	121.53 (18)	H22B—C22—H22D	71.1
C14—C9—C8	119.73 (17)	H22C—C22—H22D	107.1
C11—C10—C9	120.73 (19)	C22—C23A—H22C	38.6
C11—C10—H10	118.5 (13)	C22—C23A—H23A	109.5
C9—C10—H10	120.7 (13)	H22C—C23A—H23A	142.0
C10—C11—C12	120.61 (19)	C22—C23A—H23B	109.5
C10—C11—H11	118.6 (13)	H22C—C23A—H23B	102.8
C12—C11—H11	120.8 (13)	C22—C23A—H23C	109.5
N4—C12—C13	121.5 (2)	H22C—C23A—H23C	76.8
N4—C12—C11	119.96 (19)	C22—C23B—H23D	109.5
C13—C12—C11	118.58 (18)	C22—C23B—H23E	109.5
C12—N4—H1N4	114.5 (18)	H23D—C23B—H23E	109.5
C12—N4—H2N4	117.8 (18)	C22—C23B—H23F	109.5
H1N4—N4—H2N4	125 (3)	H23D—C23B—H23F	109.5
C14—C13—C12	120.63 (19)	H23E—C23B—H23F	109.5
C14—C13—H13	119.5 (15)		
			
O6—Fe—O2—N1	175.55 (12)	O3—C8—C9—C10	149.8 (2)
O4—Fe—O2—N1	−92.72 (12)	N3—C8—C9—C10	−29.2 (3)
O5—Fe—O2—N1	96.80 (12)	O3—C8—C9—C14	−27.4 (3)
O1—Fe—O2—N1	9.49 (11)	N3—C8—C9—C14	153.54 (19)
O3—Fe—O2—N1	−36.0 (3)	C14—C9—C10—C11	−1.0 (3)
Fe—O2—N1—C1	−11.7 (2)	C8—C9—C10—C11	−178.25 (19)
O2—N1—C1—O1	6.1 (3)	C9—C10—C11—C12	−1.7 (3)
O2—N1—C1—C2	−174.36 (16)	C10—C11—C12—N4	−177.0 (2)
N1—C1—O1—Fe	2.5 (2)	C10—C11—C12—C13	2.9 (3)
C2—C1—O1—Fe	−176.99 (14)	N4—C12—C13—C14	178.27 (19)
O2—Fe—O1—C1	−6.88 (13)	C11—C12—C13—C14	−1.6 (3)
O6—Fe—O1—C1	−62.3 (2)	C12—C13—C14—C9	−1.0 (3)
O4—Fe—O1—C1	83.59 (13)	C10—C9—C14—C13	2.3 (3)
O5—Fe—O1—C1	−111.26 (13)	C8—C9—C14—C13	179.61 (18)
O3—Fe—O1—C1	161.76 (13)	O2—Fe—O6—N5	−100.53 (12)
O1—C1—C2—C3	20.6 (3)	O4—Fe—O6—N5	166.72 (12)
N1—C1—C2—C3	−158.8 (2)	O5—Fe—O6—N5	3.73 (11)
O1—C1—C2—C7	−160.5 (2)	O1—Fe—O6—N5	−46.5 (3)
N1—C1—C2—C7	20.0 (3)	O3—Fe—O6—N5	88.00 (12)
C7—C2—C3—C4	−1.3 (3)	Fe—O6—N5—C15	−4.7 (2)
C1—C2—C3—C4	177.7 (2)	O6—N5—C15—O5	2.6 (3)
C2—C3—C4—C5	0.9 (4)	O6—N5—C15—C16	−175.01 (16)
C3—C4—C5—N2	178.1 (2)	N5—C15—O5—Fe	0.8 (2)
C3—C4—C5—C6	0.6 (4)	C16—C15—O5—Fe	178.37 (13)
N2—C5—C6—C7	−179.2 (2)	O2—Fe—O5—C15	85.37 (13)
C4—C5—C6—C7	−1.7 (4)	O6—Fe—O5—C15	−2.56 (12)
C5—C6—C7—C2	1.3 (4)	O4—Fe—O5—C15	−64.7 (2)
C3—C2—C7—C6	0.2 (4)	O1—Fe—O5—C15	164.44 (12)
C1—C2—C7—C6	−178.7 (2)	O3—Fe—O5—C15	−106.35 (12)
O2—Fe—O4—N3	166.76 (12)	O5—C15—C16—C17	18.8 (3)
O6—Fe—O4—N3	−102.43 (12)	N5—C15—C16—C17	−163.68 (18)
O5—Fe—O4—N3	−42.2 (2)	O5—C15—C16—C21	−157.92 (18)
O1—Fe—O4—N3	87.02 (12)	N5—C15—C16—C21	19.6 (3)
O3—Fe—O4—N3	0.40 (11)	C21—C16—C17—C18	1.2 (3)
Fe—O4—N3—C8	1.7 (2)	C15—C16—C17—C18	−175.66 (18)
O4—N3—C8—O3	−4.0 (3)	C16—C17—C18—C19	0.4 (3)
O4—N3—C8—C9	175.04 (16)	C17—C18—C19—N6	179.44 (18)
N3—C8—O3—Fe	4.1 (2)	C17—C18—C19—C20	−2.4 (3)
C9—C8—O3—Fe	−174.89 (14)	N6—C19—C20—C21	−178.92 (19)
O2—Fe—O3—C8	−60.9 (3)	C18—C19—C20—C21	3.0 (3)
O6—Fe—O3—C8	86.72 (13)	C19—C20—C21—C16	−1.4 (3)
O4—Fe—O3—C8	−2.41 (12)	C17—C16—C21—C20	−0.6 (3)
O5—Fe—O3—C8	164.62 (13)	C15—C16—C21—C20	176.07 (18)
O1—Fe—O3—C8	−105.33 (13)		

### Hydrogen-bond geometry (Å, °)

**Table:**

*D*—H···*A*	*D*—H	H···*A*	*D*···*A*	*D*—H···*A*
O7—H1O7···O6^i^	0.78 (3)	1.91 (3)	2.694 (2)	175 (3)
N6—H1N6···O3^ii^	0.84 (2)	2.15 (2)	2.968 (2)	163.9 (19)
N5—H1N5···O7	0.84 (2)	1.91 (2)	2.740 (2)	176 (2)
N3—H1N3···O4^iii^	0.86 (3)	1.98 (3)	2.755 (2)	150 (2)
N1—H1N1···O2^iv^	0.84 (3)	1.99 (3)	2.737 (2)	149 (2)

Symmetry codes: (i) −*x*+1, −*y*+1, −*z*; (ii) −*x*+1, −*y*+1, −*z*+1; (iii) −*x*, −*y*+1, −*z*; (iv) −*x*, −*y*, −*z*.
